# How Does Testosterone Augment the Anabolic Response to Exercise in Frail Older Adults?

**DOI:** 10.1093/geroni/igab046.1146

**Published:** 2021-12-17

**Authors:** Shalendar Bhasin

**Affiliations:** Brigham and Women's Hospital, Harvard Medical School, Boston, Massachusetts, United States

## Abstract

Testosterone treatment increases muscle mass, strength, and leg power in menopausal women, hypogonadal men, older men with mobility limitation, COPD and ESRD. Testosterone's effects on muscle mass and strength are augmented by exercise training and growth hormone. Testosterone treatment improves some measures of physical performance, such as stair climbing power and aerobic capacity; the improvements in gait speed have been modest. Testosterone increases muscle mass by inducing the hypertrophy of type 1 and 2 muscle fibers, and by increasing satellite cell number. Testosterone promotes the differentiation of mesenchymal progenitor cells into myogenic lineage and inhibits their differentiation into adipogenic lineage by activating Wnt-target genes, including follistatin that plays an important role in mediating testosterone's effects on the muscle. Testosterone also increases polyamine synthesis in the muscle. Combined administration of testosterone plus multi-component exercise intervention that includes functional training may be needed to improve function and mobility in older adults.

